# Prevalence and Determinants of Impaired Bone Mineral Density and Fractures in the First National Dutch Wilms Tumor Survivor Cohort, a National DCCSS‐LATER Study

**DOI:** 10.1002/cam4.71229

**Published:** 2025-09-16

**Authors:** Francis S. P. L. Wens, Demi T. C. de Winter, Geert O. Janssens, Rens Litjens, Jenneke E. van Atteveld, Rutger A. J. Nievelstein, Monique G. G. Hobbelink, Andrica C. H. de Vries, Jacqueline J. Loonen, Eline van Dulmen‐den Broeder, Helena J. H. van der Pal, Saskia M. F. Pluijm, Leontien C. M. Kremer, Margriet van der Heiden‐van der Loo, Marloes Louwerens, Hanneke M. van Santen, Daniel S. Olsson, Imo Hoefer, Sjoerd A. A. van den Berg, Harm van Tinteren, Sebastian J. C. M. M. Neggers, Marry M. van den Heuvel‐Eibrink

**Affiliations:** ^1^ Princess Máxima Center for Pediatric Oncology Utrecht the Netherlands; ^2^ University Medical Center Utrecht Utrecht the Netherlands; ^3^ University of Amsterdam Amsterdam the Netherlands; ^4^ Sophia Children's Hospital/Erasmus Medical Center Rotterdam the Netherlands; ^5^ Radboud University Medical Center Nijmegen the Netherlands; ^6^ Amsterdam University Medical Center Amsterdam the Netherlands; ^7^ Emma Children's Hospital/Amsterdam UMC Amsterdam the Netherlands; ^8^ Leiden University Medical Center Leiden the Netherlands; ^9^ Division of Childhealth, Wilhelmina Children's Hospital University Medical Center Utrecht the Netherlands; ^10^ Institute of Medicine University of Gothenburg Gothenburg Sweden; ^11^ Department of Clinical Chemistry Erasmus Medical Center Rotterdam the Netherlands; ^12^ Department Internal Medicine Section Endocrinology Erasmus Medical Center Rotterdam the Netherlands

**Keywords:** bone mineral density, fractures, late effects, survivorship, Wilms tumor

## Abstract

**Background:**

Wilms tumors (WT) are the most common kidney tumors in children, with excellent survival rates (90%). However, late adverse effects warrant attention. Limited data exist on musculoskeletal sequelae in WT survivors. We aimed to assess the prevalence and determinants of impaired bone mineral density (BMD) and fractures in a national cohort of Dutch WT survivors.

**Method:**

This cross‐sectional study includes WT survivors treated between 1963 and 2002, recruited as part of the DCCSS‐LATER cohort between 2016 and 2020. Dual‐energy X‐ray absorptiometry (DXA) scans were used to assess BMD. Low BMD was defined as a *Z*‐score ≤ 1. From 5 years after diagnosis, fracture prevalence was assessed by questionnaires. Univariable logistic regression was used to analyze associations between impaired BMD as well as fractures with independent variables like patient characteristics, treatments, comorbidities, and lifestyle‐related factors.

**Results:**

Of 437 invited kidney tumor survivors, 233 WT survivors participated (median age 32.1 years, median follow‐up 27.8 years). DXA scans and fracture data were available for 173 and 221 WT survivors, respectively. Low BMD at any site was observed in 26% (*n* = 46/173) of survivors and was significantly associated with treatment including ≥ 4 drugs (OR 2.76; 95% CI = 1.13–6.70). Abdominal radiotherapy doses > 30 Gy (OR 4.84; 95% CI = 1.06–22.2) were significantly associated with low lumbar spine BMD. The prevalence of fragility fractures was 16.3% (*n* = 36/221). The standardized incidence ratio (SIR) of any first fracture was 2.34 for males and 5.38 for females.

**Conclusion:**

Wilms tumor survivors treated with ≥ 4 drugs or abdominal radiotherapy (> 30 Gy) seem to be at increased risk of impaired BMD; this could indicate the need for surveillance for this subset of Wilms tumor survivors exposed to these treatment regimens in the past.

## 1 Introduction

Wilms tumors (WT) represent 5% of all childhood cancers and 75%–80% of all pediatric kidney tumors. Overall survival rates have reached nearly 90% over recent decades [1, 2, 3, 4, 5]. This indicates that a relatively high proportion of childhood cancer survivors (CCS) are represented by WT survivors (around 10%) [6, 7]. Approximately two‐thirds of these WT survivors experience chronic health conditions such as cardiovascular and endocrine disorders, at a rate comparable to that of all CCS [8, 9, 10]. These late effects raise significant concerns due to their association with increased morbidity and higher mortality among CCS [11].

Despite the awareness of late effects in WT patients, studies are limited. To date, only one large report on late effects associated with common treatment regimens in WT survivors has been published. Weil et al. reported findings from a Children's Oncology Group (COG) study, which included 2008 unilateral WT survivors. They found that morbidity among WT survivors was significantly higher compared to siblings, with a 35‐year cumulative incidence of Common Terminology Criteria for Adverse Events (CTCAE) grade 3–5 chronic health conditions (CHC) of 34.1% compared to 14.8%, respectively. Morbidity included conditions such as intestinal obstruction, kidney failure, heart failure, and gonadal dysfunction. The risks of these conditions were even higher for survivors who underwent intensified treatment, with a relative risk of grade 3–5 CHC of 1.5 for those treated with vincristine and Actinomycin D (VA) and 6.1 for those treated with ≥ 4 drugs combined with any radiotherapy [12].

So far, impaired bone mineral density (BMD) and fractures have not been studied in national, unselected WT survivor cohorts. We anticipate that at least a subset of WT survivors may have an elevated fracture risk due to previously used local, relatively high radiotherapy dosages, as well as systemic therapy that impairs BMD [13] Addressing this knowledge gap could enhance surveillance strategies and offer insights into potential interventions to prevent bone fragility in WT survivors. Therefore, in the current study, we aim to assess the prevalence and determinants for impaired BMD and fractures in the first registered national Dutch cohort of WT survivors.

## 2 Methods

### 2.1 Subjects

This cross‐sectional study focused on the survivors of WT that were included in our Dutch Childhood Cancer Survivor Study (DCCSS) LATER cohort, which included CCS diagnosed between 1963 and 2002, who had been treated at seven pediatric oncology centers in the Netherlands [6]. Patients were recruited between 2016 and 2020 and were eligible for inclusion if they had been diagnosed before the age of 19, had survived for more than five years after diagnosis, and were aged between 18 and 45 years at the time of this study. The DCCSS‐LATER BONE Study was approved by the medical research ethics committee of the Amsterdam University Medical Center (registered at toetsingonline.nl, NL35000.018.11), and written informed consent was obtained from all participants.

### 2.2 Bone Mineral Density

Dual‐energy X‐ray absorptiometry (DXA; Hologic Discovery A and Horizon A, Marlborough, MA, USA) was used and BMD of the lumbar spine (BMD_LS_), total body (BMD_TB_), and total hip (BMD_TH_) were assessed. The BMD_LS_, BMD_TB_, and BMD_TH_ were selected as endpoints for DXA measurements due to their precision and ability to reflect different aspects of skeletal health, including both trabecular and cortical bone [14]. BMD_LS_ and BMD_TB_ were measured in six clinics and BMD_TH_ in three clinics. Low and very low BMD at any site were defined as a *Z*‐score of ≤ −1 or ≤ −2, respectively, in one or more of the BMD_LS_, BMD_TB_, and BMD_TH_ measurements [15]. A *Z*‐score indicates the number of standard deviations by which the BMD deviates from the reference data matched for age and sex, as provided by the DXA manufacturer. *Z*‐scores are used in this age group because they have not yet achieved peak bone mass [15]. We excluded BMD results for patients that had osteosynthesis material in the targeted DXA area.

### 2.3 Fractures

From 5 years after diagnosis, fracture prevalence was assessed by questionnaires. This included the number, site, and year of fracture occurrence. The fractures were categorized as any fracture, long bone fracture (fracture of the lower and upper limbs, often resulting from unidentified or unknown trauma), and fragility fractures (vertebral, hip, or lower and upper limb fractures often caused by minimal trauma) [16].

### 2.4 Potential Determinants

Demographic, disease‐related, and therapy‐related data were extracted from medical records. During a single visit to the late‐effects outpatient clinic, additional data were collected, including height and body weight measurements, and a DXA scan was performed. Peripheral blood samples were subsequently collected to assess levels of thyroid‐stimulating hormone (TSH), free thyroxine (FT4), 25‐hydroxyvitamin D (25OHD), folic acid, vitamin B12, and homocysteine. Additionally, survivors completed a questionnaire addressing general health and lifestyle factors, such as diet, medication use, and physical activity [6, 17]. Thereby, standard treatment strategies for children with WT (VA = Vincristine or Dactinomycin or Vincristine and Dactinomycin, VAD = Vincristine and/or Dactinomycin and Anthracyclines, ≥ 4 drugs = Vincristine, Dactinomycin, Doxorubicin/Epirubicin + ifosfamide, etoposide, carboplatin, cyclophosphamide and/or thiotepa, and radiotherapy) were defined and categorized as potential determinants. The variables were defined as previously described based on scientific literature, manufacturer instructions, and relevant guidelines (Table S1).

### 2.5 Statistical Analysis

Descriptive statistics were used to summarize demographic, treatment‐related, comorbidity, and lifestyle‐related factors, as well as to summarize the number of DXA scans performed and fracture history. The incidence of any first fracture occurring between 1987 and 2014 was compared with sex‐adjusted and age‐adjusted population‐level fracture incidence data from a previously described Swedish national registry as Dutch population‐level data are not available. We calculated the standardized incidence ratio (SIR) of any first fracture by sex and age group using the statistical package *popEpi* [18]. The SIR reflects the proportion of observed fractures and expected fractures corrected for person‐years at risk. Univariable logistic regression analysis was pursued to identify determinants for low and very low BMD at any site, BMD_LS_, BMD_TB_, BMD_TH_, and fractures. All variables were analyzed categorically, while BMD_LS_ was also analyzed continuously, as it showed the strongest positive association with fractures [19]. Categorical variables were compared using the chi‐square test; when expected cell counts were below 5, Fisher's exact test was used. Odds ratios were reported with 95% confidence intervals, and *p*‐values were calculated. A *p*‐value < 0.05 was considered statistically significant. Due to the low number of events (i.e., low BMD outcomes or fractures) it was considered inappropriate to perform a multivariable analysis. Statistical analyses were performed using R version 4.4.0.

## 3 Results

### 3.1 Patient Characteristics

**FIGURE 1:**
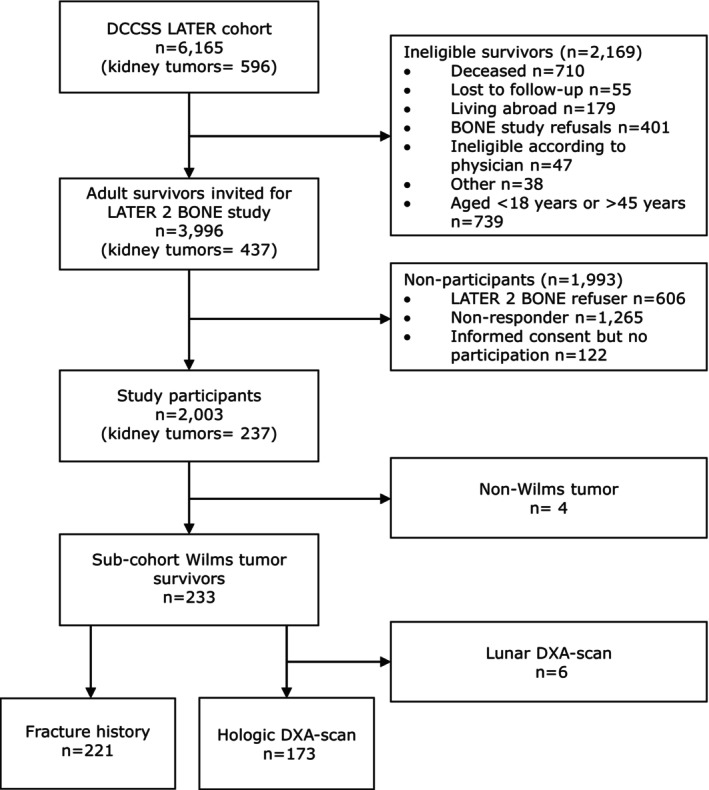
Flowchart. DCCSS, Dutch Childhood Cancer Survivor Study; DXA, dual‐energy X‐ray absorptiometry.

**FIGURE 2:**
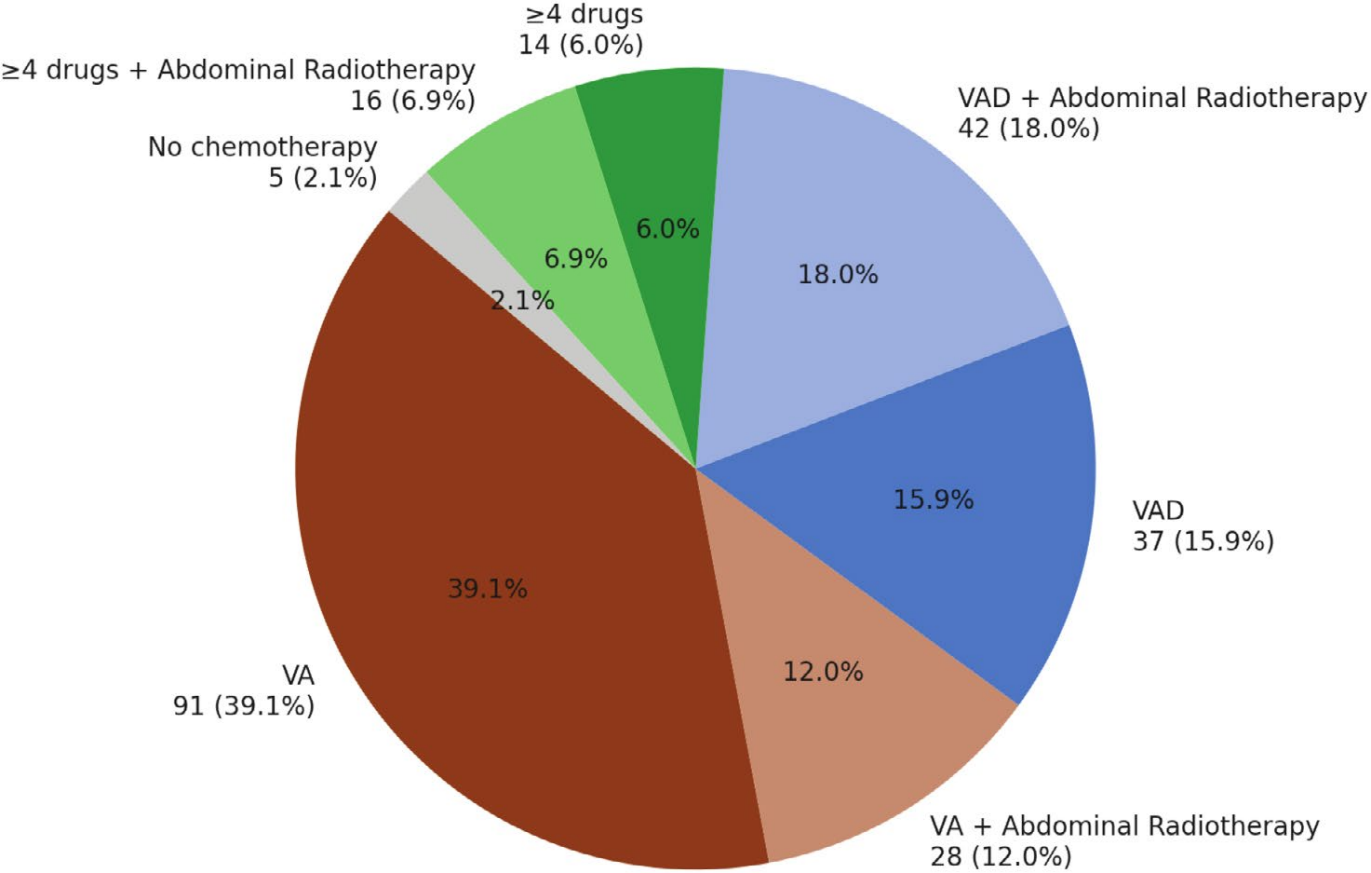
Chemotherapy and abdominal radiotherapy distribution. ≥ 4 drugs, Vincristine, Dactinomycin, Doxorubicin/Epirubicin + ifosfamide, etoposide, carboplatin, cyclophosphamide and/or thiotepa; VA, Vincristine or Dactinomycin or Vincristine and Dactinomycin; VAD, Vincristine and/or Dactinomycin and Anthracyclines.

The DCCSS‐LATER cohort consists of a total of 6165 DCCS, of which 596 were kidney tumor survivors. A total of 3996 DCCS were invited, including 437 kidney tumor survivors, and of those, 2003 survivors participated, including 233 WT survivors (Figure 1). The WT survival cohort had a mean age at diagnosis of 3.3 (standard deviation [SD] ±2.2) years, with a median age of 32.1 (interquartile range [IQR] 25.7–38.5) years at outpatient clinic visit. The median follow‐up time since diagnosis was 27.8 (IQR 21.5–34.4) years. Among all participants, 58.7% were female (Table 1). DXA scans were performed in 173 (74.2%) participants, and fracture data were available in 221 (94.8%). All WT survivors except one (0.4%) underwent nephrectomy. A total of 227 (97.4%) WT survivors received chemotherapy: 119 (51.1%) were treated with VA, 79 (33.9%) with VAD, and 30 (12.9%) with ≥ 4 drugs. Abdominal radiotherapy was administered in 87 WT survivors (37.3%): 28/119 in the VA group, 42/79 in the VAD group, and 16/30 in the ≥ 4 drugs group (Figure 2).

**TABLE 1:** Baseline characteristics of participants.

	Participants (*n* = 233)	Participants with eligible DXA‐scan (*n* = 173)	Participants with data on fractures (*n* = 221)
Sex			
Male	96 (41.2)	71 (41.0)	87 (39.4)
Female	137 (58.7)	102 (59.0)	134 (60.6)
Age at invitation (yr.)			
10–19	8 (3.4)	8 (4.6)	8 (3.6)
20–29	92 (39.5)	71 (41.0)	87 (39.4)
30–39	91 (39.1)	62 (35.8)	86 (38.9)
40–45	42 (18.0)	32 (18.5)	40 (18.1)
Age at diagnosis (yr.)			
0–5	180 (77.3)	135 (78.0)	171 (77.4)
5–10	51 (21.9)	38 (22.0)	48 (21.7)
10–15	2 (0.01)	0 (0.0)	2 (0.9)
Follow‐up since childhood cancer diagnosis (yr.)			
0–9	NA	NA	NA
10–19	44 (18.9)	33 (19.1)	43 (19.5)
20–29	90 (38.6)	69 (39.9)	84 (38.0)
30–39	72 (30.9)	50 (30.0)	69 (31.2)
40–49	27 (11.6)	21 (12.1)	25 (11.3)
BMI^a^			
Underweight	7 (3.0)	6 (3.5)	6 (2.7)
Normal	133 (57.1)	101 (58.4)	128 (57.9)
Overweight/obese	88 (37.8)	65 (37.6)	87 (39.4)
Smoking			
Never	128 (54.9)	94 (54.3)	125 (56.6)
Current	28 (12.0)	24 (13.9)	23 (10.4)
Former	36 (15.5)	25 (14.5)	35 (15.8)
Heavy drinking^b^			
Yes	7 (3.0)	5 (2.9)	7 (3.2)
No	197 (84.5)	143 (82.7)	187 (84.6)
Low physical activity^c^			
Yes	27 (11.6)	21 (12.1)	27 (12.2)
No	166 (71.2)	128 (74.0)	158 (71.5)
Low calcium intake (< 3500 g/week)			
Yes	52 (22.3)	36 (20.8)	51 (23.1)
No	139 (59.7)	111 (64.2)	132 (59.7)
Low eGFR (< 90 mL/min/1.73 m^2^)			
Yes	153 (65.7)	121 (69.9)	149 (67.4)
No	72 (30.9)	50 (28.9)	70 (31.7)
Vitamin D deficiency (< 50 nmol/L)			
Yes	65 (27.9)	50 (28.9)	63 (28.5)
No	160 (68.7)	121 (69.9)	156 (70.6)
Elevated homocysteine levels (> 19 μmol/L)			
Yes	25 (10.7)	20 (11.6)	23 (10.4)
No	200 (85.8)	151 (87.3)	196 (88.7)
Vitamin B12 deficiency (< 150 nmol/L)			
Yes	11 (4.7)	8 (4.6)	10 (4.5)
No	214 (91.8)	163 (94.2)	209 (94.6)
Folic acid deficiency (< 6.8 nmol/L)			
Yes	33 (14.2)	23 (13.3)	31 (14.0)
No	192 (84.2)	148 (85.5)	188 (85.1)
Chemotherapy			
VA	119 (51.1)	90 (52.0)	109 (49.3)
VAD	79 (33.9)	54 (31.2)	77 (34.8)
≥ 4 drugs	30 (12.9)	24 (13.9)	30 (13.6)
No chemotherapy	5 (2.1)	5 (2.9)	5 (2.3)
Abdominal radiotherapy			
Yes	87 (37.3)	62 (35.8)	84 (38.0)
No	146 (62.7)	111 (6.1)	137 (62.0)
Abdominal radiotherapy + chemotherapy			
Abdominal RT + VA	28 (32.2)	21 (33.9)	26 (31.0)
Abdominal RT + VAD	42 (48.3)	28 (45.2)	41 (48.8)
Abdominal RT + ≥ 4 drugs	16 (18.4)	12 (19.4)	16 (19.0)
Abdominal RT + none	1 (1.1)	1 (1.6)	1 (1.2)
Nephrectomy			
Yes	232 (99.6)	172 (99.4)	220 (99.5)
No	1 (0.4)	1 (0.6)	1 (0.5)

Abbreviations: ≥ 4 drugs, Vincristine, Dactinomycin, Doxorubicin/Epirubicin + ifosfamide, etoposide, carboplatin, cyclophosphamide and/or thiotepa; RT, radiotherapy; VA, Vincristine or Dactinomycin or Vincristine and Dactinomycin; VAD, Vincristine and/or Dactinomycin and Anthracyclines; yr., year.

^a^ Underweight: BMI < 18.5 kg/m^2^; Normal: BMI ≥ 18.5 and < 25 kg/m^2^; Overweight: BMI ≥ 25 to < 30 kg/m^2^; Obese: BMI ≥ 30 kg/m^2^.

^b^ Males: > 14 alcoholic consumptions per week; Females: > 7 alcoholic consumptions per week (self‐report).

^c^ Low physical activity = < 20th percentile.

### 3.2 Prevalence of Impaired BMD

Low BMD at any site was observed in 46/173 WT survivors (26.6%; 95% CI = 20.6%–33.6%). Low BMD_LS_, BMD_TB_, and BMD_TH_ were observed in 19.8% (95% CI = 14.5%–26.4%), 15.1% (95% CI = 10.4%–21.3%), and 12.3% (95% CI = 6.6%–21.8%), respectively. Wilms tumor survivors treated with VA had low BMD at any site in 19.1% (95% CI = 12.3%–28.5%), compared to 29.6% (95% CI = 19.1%–42.8%) in those treated with VAD and 45.8% (95% CI = 27.9%–64.9%) in those treated with ≥ 4 drugs. Among survivors who received abdominal radiotherapy (mean dose: 23.34 Gy, range: 6.45–60.0 Gy), the prevalence of low BMD at any site was 30.6% (95% CI = 20.6%–43.0%).

BMD at any site was scored very low in 9 WT survivors (5.2%; 95% CI = 2.8%–9.6%). Very low BMD_LS,_ BMD_TB_, and BMD_TH_ were observed in 4.7% (95% CI = 2.4%–8.9%), 0.6% (95% CI = 0.1%–3.3%), and 1.4% (95% CI = 0.2%–7.4%) respectively. Very low BMD at any site was found in 5.6% (95% CI = 2.4%–12.5%) for WT survivors that had been treated with VA, 3.7% (95% CI = 1.0%–12.5%) for VAD, 8.3% (95% CI = 2.3%–25.8%) for ≥ 4 drugs, and 6.5% (95% CI = 2.5%–15.4%) in WT survivors treated with abdominal radiotherapy.

### 3.3 Prevalence of Fractures

A total of 70/221 (31.7%) WT survivors reported at least one fracture, with fractures categorized as any fracture 31.7% (95% CI = 25.9%–38.1%), long bone fractures 21.7% (95% CI = 16.8%–27.6%), and fragility fractures 16.3% (95% CI = 12.0%–21.7%). The SIR of any fracture was 2.34 (95% CI = 1.07–3.62) for male participants and 5.38 (95% CI = 3.08–7.68) for female participants as compared to the Swedish reference cohort. Among WT survivors treated with VA, 34.3% (95% CI = 26.0%–43.6%) experienced any fracture, compared to 24.7% (95% CI = 16.4%–35.4%) treated with VAD, 40.0% (95% CI = 24.6%–57.7%) treated with ≥ 4 drugs, and 31.0% (95% CI = 22.1%–41.2%) in those who received abdominal radiotherapy.

### 3.4 Determinants for Impaired BMD

Univariable analysis revealed significant associations between low BMD at any site and prior treatment with ≥ 4 drugs (OR 2.76; 95% CI = 1.13–6.70), being underweight (OR 10.78; 95% CI = 1.21–96.10), and overweight/obese (OR 0.35; 95% CI = 0.15–0.79). Low BMD_LS_ was associated with abdominal radiotherapy doses > 30 Gy (OR 4.84; 95% CI = 1.06–22.2); however, when analyzed as a continuous variable, abdominal radiotherapy dose was not significantly associated. Additionally, low BMD_LS_ was significantly associated with being underweight (OR 16.96; 95% CI = 1.89–152.22) and overweight/obese (OR 0.35; 95% CI = 0.12–0.87). Low BMD_TB_ was associated with prior treatment with ≥ 4 drugs (OR 3.04; 95% CI = 1.10–8.40) (Table 2 and Table S2).

**TABLE 2:** Patient and treatment‐related risk factors for low BMD outcomes (*Z*‐score ≤ −1) using univariable logistic regression analysis.

	Low BMD at any site (*n* = 46/173)			Low lumbar spine BMD (*n* = 34/172)			Low total body BMD (*n* = 25/166)			Low total hip BMD (*n* = 9/73)		
	No.	OR (95% CI)	*p* *	No.	OR (95% CI)	*p* *	No.	OR (95% CI)	*p* *	No.	OR (95% CI)	*p* *
Patient characteristics												
Sex		1.46 (0.74–2.89)	0.28		1.80 (0.85–3.84)	0.13		0.87 (0.37–2.08)	0.76		NA	0.72
Male	22/71 (31.0)			18/71 (25.4)			10/71 (14.1)			3/71 (4.23)		
Female	24/87 (27.6)			16/87 (18.4)			15/87 (17.2)			6/87 (6.90)		
Attained age (per year)												
18–25	13/48 (27.1)	Ref		10/48 (20.8)	Ref		7/46 (15.2)	Ref		3/19 (15.8)	Ref	
26–35	18/65 (27.7)	1.03 (0.44–2.38)	0.94	12/65 (18.5)	0.86 (0.34–2.20)	0.75	10/65 (15.4)	1.01 (0.35–2.89)	0.98	2/26 (7.69)	0.44 (0.07–2.96)	0.40
> 36	15/60 (25.0)	0.90 (0.38–2.13)	0.81	12/59 (20.3)	0.97 (0.38–2.49)	0.95	8/55 (5.68)	0.95 (0.32–2.85)	0.93	4/28 (14.3)	0.89 (0.18–4.51)	0.89
Age at diagnosis (per year)												
0–5	32/135 (23.7)	Ref		24/134 (17.9)	Ref		18/128 (14.1)	Ref		8/58 (13.8)	Ref	
5–10^a^	14/38 (36.8)	1.88 (0.87–4.05)	0.11	10/38 (26.3)	1.64 (0.70–3.82)	0.25	7/38 (18.4)	1.38 (0.53–3.60)	0.51	1/15 (6.67)	0.45 (0.05–3.88)	0.47
BMI^b^												
Underweight	5/6 (83.3)	10.78 (1.21–96.10)	**0.033**	5/6 (83.3)	16.96 (1.89–152.55)	**0.012**	2/5 (40.0)	NA	NA	1/2 (50.0)	NA	NA
Normal	32/101 (31.7)	Ref		23/101 (22.8)	Ref		19/97 (19.6)	NA	NA	7/44 (15.9)	NA	NA
Overweight or obese	9/65 (13.8)	0.35 (0.15–0.79)	**0.011**	6/64 (9.38)	0.35 (0.12–0.87)	**0.033**	4/63 (6.35)	NA	NA	1/26 (3.85)	NA	NA
Follow‐up time (per year)												
10–19	9/33 (27.3)	Ref		6/33 (18.2)	Ref		6/32 (18.8)	Ref		3/13 (23.1)	Ref	
20–29	21/69 (30.4)	1.17 (0.46–2.93)	0.74	15/69 (21.7)	1.25 (0.44–3.58)	0.68	10/68 (14.7)	0.75 (0.25–2.27)	0.61	1/26 (3.85)	0.13 (0.01–1.44)	0.097
30–39	10/50 (20.0)	0.67 (0.24–1.87)	0.44	8/50 (16.0)	0.86 (0.27–2.74)	0.80	6/49 (12.2)	0.60 (0.18–2.07)	0.42	3/26 (11.5)	0.43 (0.07–2.54)	0.35
40–49	6/21 (28.6)	1.07 (0.32–3.61)	0.91	5/20 (25.0)	1.50 (0.39–5.75)	0.55	3/17 (17.6)	0.93 (0.20–4.29)	0.92	2/8 (25.0)	1.11 (0.14–8.68)	0.92
Treatment characteristics												
VA		0.43 (0.22–0.87)	**0.020**		0.51 (0.23–1.09)	0.08		0.59 (0.25–1.39)	0.23		NA	1.0
Yes	17/90 (18.9)			13/89 (14.6)			10/85 (11.8)			4/34 (11.8)		
No	29/83 (34.9)			21/83 (25.3)			15/81 (18.5)			5/39 (12.8)		
VAD		1.25 (0.61–2.56)	0.5		1.25 (0.57–2.75)	0.56		1.00 (0.40–2.50)	0.99		NA	0.26
Yes	16/54 (29.6)			12/54 (22.2)			8/53 (15.1)			5/25 (20.0)		
No	30/119 (25.2)			22/118 (18.6)			17/113 (15.4)			4/48 (8.33)		
≥ 4 drugs		2.76 (1.13–6.70)	**0.030**		1.85 (0.70–4.89)	0.22		3.04 (1.10–8.40)	**0.030**		NA	NA
Yes	11/24 (45.8)			7/24 (29.2)			7/23 (30.4)			0/11 (0.00)		
No	35/149 (23.5)			27/148 (18.2)			18/143 (12.6)			9/62 (14.5)		
RT abdominal		1.37 (0.69–2.75)	0.37		1.58 (0.74–3.39)	0.24		1.57 (0.66–3.73)	0.31		NA	1.0
Yes	19/62 (30.6)			15/61 (24.6)			11/58 (19.0)			4/29 (13.8)		
No	27/111 (24.3)			19/111 (17.1)			14/108 (13.0)			5/44 (11.4)		
Abdominal RT dose												
0 Gy	27/111 (24.3)	REF	REF	19/111 (17.1)	REF	REF	14/108 (13.0)	REF	REF	5/44 (11.4)	NA	NA
0–30 Gy	15/54 (27.8)	1.20 (0.56–2.48)	0.63	11/53 (20.8)	1.27 (0.54–2.87)	0.57	8/50 (16.0)	1.27 (0.48–3.22)	0.61	4/28 (16.0)	NA	
> 30 Gy	4/8 (50.0)	3.11 (0.69–14.0)	0.13	4/8 (50.0)	4.84 (1.06–22.2)	**0.036**	3/8 (37.5)	4.02 (0.76–18.4)	0.076	0/1 (0.00)	NA	
RT thorax		NA	1.0		NA	1.0		NA	0.42		NA	0.81
Yes	4/14 (28.6)			3/14 (21.4)			3/13 (23.1)			1/10 (10.0)		
No	42/159 (26.4)			31/158 (19.6)			22/153 (14.4)			8/63 (12.7)		

*Note:* Bold values indicate statistical significance at *p* < 0.05.

Abbreviations: ≥ 4 drugs, Vincristine, Dactinomycin, Doxorubicin/Epirubicin + ifosfamide, etoposide, carboplatin, cyclophosphamide and/or thiotepa; BMD, bone mineral density; BMI, body mass index; CI, confidence interval; Gy, Gray; NA, not applicable (due to insufficient patient numbers < 5); No., number; OR, odds ratio; Ref, reference; RT, radiotherapy; VA, Vincristine or Dactinomycin or Vincristine and Dactinomycin; VAD, Vincristine and/or Dactinomycin and Anthracyclines.

* Logistic regression *p*‐value for variables with more than five observations in each cell. For variables with less than five observations in each cell, a Fisher exact *p*‐value was calculated.

^a^ No patients in the 10–15‐year group.

^b^ Adjusted for amputation.

Lifestyle‐related factors including current smoking (OR 2.08; 95% CI = 0.82–5.30), low dietary calcium intake (OR 1.14; 95% CI = 0.50–2.58), and low physical activity (OR 2.07; 95% CI = 0.80–5.36) were positive, although not significantly associated with low BMD at any site.

Similarly, Vitamin D deficiency (OR 1.13; 95% CI = 0.54–2.37), elevated homocysteine levels (OR 2.05; 95% CI = 0.78–5.41), and folic acid deficiency (OR 1.27; 95% CI = 0.48–3.31) showed higher odds without statistical significance (Table 4). In the limited number of participants with very low BMD, no significant associations could be determined.

### 3.5 Determinants for Fractures

Low BMD_LS_ was, if any, moderately associated with the risk of any fractures (OR 2.12; 95% CI = 0.95–4.73), long bone fractures (OR 2.11; 95% CI = 0.89–5.04), and fragility fractures (OR 1.91; 95% CI = 0.72–5.08). When examining BMD_LS_ as a continuous *Z*‐score, the odds for any fractures showed a slight increase (OR 1.21; 95% CI = 0.92–1.60) (Table 3). Former smoking was the only determinant that was significantly associated with the occurrence of any fractures (OR 2.33; 95% CI = 1.08–5.03). A low eGFR increased the odds for any fracture (OR 1.19; 95% CI = 0.64–2.21), although not significant (Table 4 and Table S3).

**TABLE 3:** Patient and treatment‐related risk factors for fracture outcomes using univariable logistic regression analysis.

	Any fractures (*n* = 70/221)			Long bone fractures (*n* = 48/221)			Fragility fractures (*n* = 36/221)		
	No.	OR (95% CI)	*p* *	No.	OR (95% CI)	*p* *	No.	OR (95% CI)	*p* *
Patient characteristics									
Sex		1.35 (0.76–2.40)	0.31		0.90 (0.47–1.75)	0.77		0.73 (0.35–1.56)	0.42
Male	31/87 (35.6)			18/87 (20.7)			12/87 (13.8)		
Female	39/134 (29.1)			30/134 (22.4)			24/134 (17.9)		
Attained age (per year)									
18–25	21/59 (30.0)	Ref		13/59 (22.0)	Ref		10/59 (16.9)	Ref	
26–35	26/82 (37.1)	1.19 (0.59–2.42)	0.63	19/82 (23.2)	1.07 (0.48–2.38)	0.87	13/82 (15.9)	0.92 (0.37–2.28)	0.86
> 36	23/80 (32.9)	1.37 (0.67–2.81)	0.39	16/80 (20.0)	0.88 (0.39–2.01)	0.77	13/80 (16.3)	0.95 (0.39–2.35)	0.91
Age at diagnosis (per year)									
0–5	54/171 (31.6)	Ref		36/171 (21.2)	Ref		27/171 (15.8)	Ref	
5–10^a^	16/48 (33.3)	0.92 (0.48–1.82)	0.82	12/48 (25.0)	1.25 (0.59–2.65)	0.56	9/48 (18.8)	1.23 (0.53–2.83)	0.63
BMI^b^									
Underweight	4/6 (66.7)	NA	NA	3/6 (50.0)	NA	NA	3/6 (50.0)	NA	NA
Normal	34/128 (26.6)	Ref		21/128 (16.4)	Ref		16/128 (12.5)	Ref	
Overweight/obese	32/87 (36.8)	NA	NA	24/87 (27.6)	NA	NA	17/87 (19.5)	NA	NA
Follow‐up time (per year)									
10–19	16/43 (37.2)	Ref		10/43 (23.3)	Ref		8/43 (18.6)	Ref	
20–29	29/84 (34.5)	0.89 (0.41–1.91)	0.76	21/84 (25.0)	1.10 (0.46–2.60)	0.82	13/84 (15.5)	0.80 (0.30–2.11)	0.65
30–39	19/69 (27.5)	0.64 (0.28–1.45)	0.28	11/69 (15.9)	0.63 (0.24–1.63)	0.34	9/69 (13.0)	0.66 (0.23–1.86)	0.43
40–49	6/25 (24.0)	0.53 (0.18–1.61)	0.27	6/25 (24.0)	1.04 (0.33–3.32)	0.94	6/25 (24.0)	1.38 (0.42–4.57)	0.60
Treatment characteristics									
VA		1.23 (0.70–2.17)	0.47		1.23 (0.70–2.17)	0.47		1.54 (0.75–3.18)	0.24
Yes	37/109 (33.9)			28/109 (25.7)			21/109 (19.3)		
No	33/112 (29.5)			20/112 (17.9)			15/112 (13.4)		
VAD		0.60 (0.32–1.11)	0.10		0.42 (0.19–0.89)	**0.02**		0.57 (0.25–1.29)	0.18
Yes	19/77 (24.7)			10/77 (13.0)			9/77 (11.7)		
No	51/144 (35.4)			38/144 (26.4)			27/144 (18.8)		
≥ 4 drugs		1.53 (0.69–3.38)	0.29		1.37 (0.57–3.31)	0.48		NA	0.79
Yes	12/30 (40.0)			8/30 (26.7)			4/30 (13.3)		
No	58/191 (30.4)			40/191 (20.9)			32/191 (16.8)		
RT abdominal		0.95 (0.53–1.70)	0.86		0.77 (0.39–1.51)	0.45		0.78 (0.37–1.67)	0.53
Yes	26/84 (31.0)			16/84 (19.0)			12/84 (14.3)		
No	44/137 (32.1)			32/137 (23.4)			24/137 (17.5)		
Abdominal RT dose									
0 Gy	44/137 (32.1)	REF	REF	32/137 (23.3)	REF	REF	24/137 (17.5)	REF	REF
0–30 Gy	6/13 (46.2)	1.21 (0.65–2.29)	0.56	2/13 (15.4)	0.81 (0.39–1.61)	0.55	2/13 (15.4)	0.77 (0.33–1.68)	0.53
> 30 Gy	20/71 (28.2)	0.55 (0.17–1.81)	0.31	14/71 (19.7)	0.60 (0.09–2.37)	0.52	10/71 (14.1)	0.86 (0.13–3.46)	0.85
RT thorax		NA	0.44		NA	NA		NA	NA
Yes	4/18 (22.2)			0/18 (0.00)			0/18 (0.00)		
No	66/203 (32.5)			48/203 (23.6)			36/203 (17.7)		
Low BMD									
Low BMD at any site		1.68 (0.81–3.47)	0.16		1.45 (0.64–3.27)	0.38		1.16 (0.45–2.99)	0.77
Yes	17/43 (39.5)			11/43 (25.6)			7/43 (16.3)		
No	35/125 (28.0)			24/125 (19.2)			18/125 (14.4)		
Low BMD_LS_		2.12 (0.95–4.73)	0.07		2.11 (0.89–5.04)	0.09		1.91 (0.72–5.08)	0.19
Yes	14/31 (45.2)			10/31 (32.3)			7/31 (22.6)		
No	38/136 (27.9)			25/136 (18.4)			18/136 (13.2)		
Low BMD_LS, continuous *Z*‐score_		1.21 (0.92–1.60)	0.17		0.87 (0.63–1.18)	0.36		0.79 (0.55–1.12)	0.19
Low BMD_TB_		1.73 (0.71–4.22)	0.23		1.36 (0.49–3.75)	0.56		NA	0.75
Yes	10/24 (41.7)			6/24 (25.0)			4/24 (16.7)		
No	40/137 (29.2)			27/137 (19.7)			19/137 (13.9)		
Low BMD_TH_		NA	1		NA	NA		NA	NA
Yes	2/9 (22.2)			0/9 (0.00)			0/9 (0.00)		
No	19/64 (29.7)			13/64 (20.3)			10/64 (15.6)		
Very low BMD at any site		NA	1		NA	0.64		NA	0.28
Yes	2/7 (28.6)			2/7 (28.6)			2/7 (28.6)		
No	50/161 (31.1)			33/161 (20.5)			23/161 (14.3)		

Abbreviations: ≥ 4 drugs, Vincristine, Dactinomycin, Doxorubicin/Epirubicin + ifosfamide, etoposide, carboplatin, cyclophosphamide and/or thiotepa; BMD, bone mineral density; BMI, body mass index; CI, confidence interval; Gy, Gray; LS, lumbar spine; NA, not applicable (due to insufficient patient numbers < 5); No., number; OR, odds ratio; Ref, reference; RT, radiotherapy; TB, total body; TH, total hip; VA, Vincristine or Dactinomycin or Vincristine and Dactinomycin; VAD, Vincristine and/or Dactinomycin and Anthracyclines.

* Logistic regression *p*‐value for variables with more than five observations in each cell. For variables with less than five observations in each cell, a Fisher exact *p*‐value was calculated.

^a^ No patients in the 10–15‐year group.

^b^ Adjusted for amputation.

**TABLE 4:** Comorbidities, lifestyle, and metabolic‐related risk factors for low BMD (*Z*‐score ≤ −1) and fracture outcomes using univariable logistic regression analysis.

	Low BMD at any site (*n* = 46/173)			Any fractures (*n* = 70/221)		
	No.	OR (95% CI)	*p* *	No.	OR (95% CI)	*p* *
Comorbidities						
Hyperthyroidism		NA	0.17		NA	NA
Yes	2/3 (66.7)			NA		
No	43/168 (25.6)			NA		
Low eGFR		0.89 (0.42–1.86)	0.75		1.19 (0.64–2.21)	0.59
Yes	31/121 (25.6)			48/149 (32.3)		
No	14/50 (28.0)			20/70 (28.6)		
Lifestyle variables						
Smoking						
Never	24/94 (25.5)	Ref	Ref	36/125 (28.9)	Ref	Ref
Former	4/25 (16.0)	NA	0.43	17/18 (94.4)	2.33 (1.08–5.03)	**0.030**
Current	10/24 (41.7)	2.08 (0.82–5.30)	0.12	8/15 (53.3)	1.32 (0.51–3.38)	0.56
Heavy drinking		NA	0.11		NA	0.23
Yes	3/5 (60.0)			4/7 (57.0)		
No	36/143 (25.2)			61/187 (32.6)		
Low dietary calcium intake		1.14 (0.50–2.58)	0.76		0.89 (0.44–1.81)	0.75
Yes	11/36 (30.6)			15/51 (29.4)		
No	31/111 (27.9)			42/132 (31.8)		
Low physical activity		2.07 (0.80–5.36)	0.13		0.41 (0.15–1.15)	0.09
Yes	9/21 (42.9)			5/27 (18.5)		
No	34/128 (26.6)			56/158 (35.4)		
Vitamin D deficiency		1.13 (0.54–2.37)	0.75		0.94 (0.50–1.78)	0.86
Yes	14/50 (28.0)			19/63 (30.2)		
No	31/121 (25.6)			49/156 (31.4)		
Severe vitamin D deficiency		1.19 (0.39–3.58)	0.76		NA	0.07
Yes	5/17 (29.4)			2/19 (10.5)		
No	40/154 (26.0)			66/200 (33.0)		
Elevated homocysteine levels		2.05 (0.78–5.41)	0.15		0.76 (0.29–2.03)	0.59
Yes	8/20 (40.0)			6/23 (26.1)		
No	37/151 (24.5)			62/196 (31.6)		
Vitamin B12 deficiency		NA	0.21		NA	1.0
Yes	4/8 (50.0)			3/10 (30.0)		
No	41/63 (65.1)			65/209 (31.1)		
Folic acid deficiency		1.27 (0.48–3.31)	0.63		1.26 (0.57–2.81)	0.57
Yes	7/23 (30.4)			11/31 (35.5)		
No	38/148 (25.7)			57/188 (30.3)		

Abbreviations: BMD, bone mineral density; CI, confidence interval; NA, not applicable (due to insufficient patient numbers < 5); No., number; OR, odds ratio.

* Logistic regression *p*‐value for variables with more than five observations in each cell. For variables with less than five observations in each cell, a Fisher exact *p*‐value was calculated.

## 4 Discussion

This national study, which described the first cohort of Dutch WT patients treated in the Netherlands, investigated bone health in relation to commonly applied treatment regimens, a topic that has not been explored in previous global cohorts. Our findings showed that WT survivors treated with ≥ 4 drugs had an increased risk for impaired BMD. At a median age of 32.1 years, 45.8% of intensively treated WT survivors revealed impaired BMD, compared to 19.1% treated with VA and 29.6% treated with VAD. This impaired BMD rate even exceeds the 42% observed in postmenopausal women aged over 50 in the general population, as reported in a systematic review and meta‐analysis of 343,704 participants from 37 countries [20].

Even though this is a relatively small cohort, this indicates that intensively treated WT survivors deserve attention and surveillance for BMD decline similar to survivors of, for instance, ALL, CNS tumors, and after SCT treatment for various indications [21, 22].

Why treatment factors contribute to this more pronounced impaired BMD may be explained by the addition of anthracyclines, doxorubicin. This is consistent with previously documented negative effects on bone strength in 68 healthy rats treated with doxorubicin or saline for seven weeks [23]. Doxorubicin induces reactive oxygen radicals by suppressing the differentiation of osteoblasts, which are important for new bone tissue formation [24]. Additionally, of the intensively treated survivors, 60% (18/30) were treated with ifosfamide. Ifosfamide affects bone indirectly through altered renal function [25]. This renal toxicity leads to hypophosphatemia, which eventually results in defective mineralization and demineralization of bone and inhibits bone formation [26]. Both doxorubicin and ifosfamide were significantly associated with a BMD decline. Alternatively, impaired BMD can be due to gonadal damage related to alkylating agents [27]. Chemaitilly et al. reported that in 921 CCS with a median follow‐up of 24 years, 10.9% developed premature ovarian insufficiency (POI). Higher cyclophosphamide equivalent dosages (CED scores) ≥ 8000 mg/m^2^ were significantly associated with the development of POI. POI is associated with impaired BMD, with survivors showing 5.5 times increased odds of impaired BMD [28]. In our setting, the cumulative dose of alkylating agents did not affect the risk of impaired BMD. This could be explained by the fact that only eight survivors in our cohort were treated with CED scores ≥ 8000 mg/m^2^. Survivors treated with ≥ 4 drugs included patients with multiple relapses or high‐risk patients who frequently receive combined treatment with radiotherapy, further contributing to BMD decline. Among the survivors treated with abdominal radiotherapy, 24.6% had an impaired BMD of the lumbar spine. Treatment with doses exceeding 30 Gy was significantly associated with a more than fourfold increased risk of impaired BMD in the lumbar spine. This finding aligns with existing in vivo studies conducted in rats and rabbits. These studies describe that higher doses of radiotherapy (> 30 Gy) were associated with reduced BMD, strength, and elasticity while increasing fragility and susceptibility to fractures [29, 30]. It is important to note that while DXA scans measure BMD, they primarily assess bone quantity and may not detect changes in bone quality caused by radiotherapy. Therefore, the impact of radiotherapy on bone health might be underestimated by DXA scans alone [31].

Moreover, we observed an increased fracture rate among WT survivors compared to Swedish population‐level data. Interestingly, Sweden has the highest reported fracture incidence worldwide, suggesting that the observed increase in our cohort may even be underestimated [32, 33]. Of particular concern is the prevalence of fragility fractures in our cohort. Fragility fractures are clinically significant as they often reflect underlying osteoporosis. In our study, 16.3% of WT survivors experienced fragility fractures at a median age of 32.1 years. This prevalence is comparable to the 17.7% reported in a cohort of 8904 individuals aged 70 years and older [34]. Despite the high fracture prevalence of 40% among intensively treated WT survivors, no significant associations were observed between treatment and fracture risk.

While treatment‐related factors for Wilms tumor are important contributors to impaired BMD, patient characteristics may also influence bone health. Our study observed a significant impairment in BMD among survivors who were underweight, and a protection among survivors who were overweight/obese at the time of recruitment. The relationship between adipose tissue and bone strength is complex, influenced by fat distribution and secretory profiles, as shown in previous studies [35, 36]. This highlights the multifactorial nature of BMD and the need for considering both treatment and patient characteristics in understanding bone health in WT survivors. The only factor at the time of recruitment that was significantly associated with the occurrence of any fractures was former smoking, and not hormones or vitamin status. Kidney function seemed to influence the odds for any fracture, but this was not a strong association in this modest sample. A potential biological explanation for how smoking (or former smoking) may contribute to reduced BMD and increased fracture risk is through mechanisms such as the effects of nicotine and cadmium on decreased PTH secretion, reduced calcium absorption, and lowered vitamin D levels [37, 38].

In this study, low BMD_LS_ appears to be the strongest indicator of fracture risk across all fracture categories. This finding is consistent with results from the full DCCSS‐LATER cohort that included 2003 survivors of all tumor types, as described by van Atteveld et al., and the study by Alarkawi et al., which assessed low BMD_LS_ as an indicator of fracture risk for men and women in the general population [19, 39]. The BMD_LS_ represents the metabolically active trabecular bone, which may be particularly affected by the intensive cancer treatment. The same observation is made in BMD_TB_, which especially reflects overall bone mass and body composition, thereby making it a valuable tool for evaluating general osteoporosis risk. The BMD_TH_ is better suited for evaluating cortical bone and its strength [14]. It should be noted that in our study, these associations did not reach statistical significance, even when lumbar spine BMD was analyzed as a continuous variable, which may be due to limited power.

Our study has some potential limitations. First, selection bias may be present, as 233 Wilms tumor survivors participated out of the 473 invited kidney tumor survivors in the LATER 2 study. Unfortunately, no detailed patient characteristic data are available for the nonparticipants, which makes it difficult to compare the baseline characteristics between participants and nonparticipants. However, Feijen et al. previously investigated participation in the DCCSS‐LATER 2 cohort study and found no significant difference in the likelihood of participation between kidney tumor survivors in a univariate analysis (*p* = 0.06) [6]. This suggests that the inclusion of kidney tumor survivors in our study may not have been disproportionately influenced by the presence of late effects, which could reduce the risk of selection bias. Second, although this is the first national cohort study exploring BMD and fractures in Wilms tumor survivors, the relatively small sample size and incidence of events limited the ability to perform reliable multivariable analysis. Third, fracture history was assessed retrospectively and that part of the fractures might have occurred before assessment of potential determinants, and therefore we cannot unravel the relationships between smoking and fractures, and BMD and fractures. Another limitation is that the method used to characterize fractures (i.e., based on medical history) may be prone to recall bias. While most reported fractures were radiographically confirmed according to the participants, we did not have access to the actual radiographs. Lastly, data on the level of trauma prior to the fractures was not available. We attempted to mitigate this effect by classifying fractures as “long bone fractures” and “fragility fractures.” Future research, including multi‐institutional cohort studies may validate the results after increasing the power of the analysis, thereby confirming the independent value of the potential determinants of impaired BMD and fractures in WT survivors.

In conclusion, our data suggest that a subset of WT survivors, particularly those who received intensive treatment with ≥ 4 drugs and/or a relatively high‐dose abdominal radiotherapy, appears to be at an elevated risk of impaired bone density and fractures. Larger cohorts with the ability to conduct multivariable analyses are necessary to confirm our findings, as these results may ultimately impact recommendations towards active surveillance. For instance, recommending at least one DXA scan upon reaching adulthood in intensively treated WT survivors could be considered, as is currently done for other CCS who are at risk [22]. Additional research and international collaboration are necessary to explore the underlying mechanisms, identify modifiable factors that may further guide the development of such surveillance recommendations, as well as future preventive and therapeutic strategies. Both SIOP‐RTSG and COG have already made serious attempts over the past decades to reduce the intensity of radiotherapy and chemotherapy in their trial designs for children with renal tumors [40, 41, 42, 43].

## Author Contributions

**Francis S. P. L. Wens:** conceptualization, methodology, investigation, validation, formal analysis, visualization, writing – original draft, writing – review and editing. **Demi T. C. de Winter:** validation, writing – review and editing. **Geert O. Janssens:** conceptualization, methodology, data curation, investigation, validation, formal analysis, supervision, visualization, project administration, resources, writing – original draft, writing – review and editing. **Rens Litjens:** validation, writing – review and editing. **Jenneke E. van Atteveld:** data curation, validation, writing – review and editing, resources. **Rutger A. J. Nievelstein:** data curation, validation, writing – review and editing, resources. **Monique G. G. Hobbelink:** data curation, validation, resources, writing – review and editing. **Andrica C. H. de Vries:** data curation, validation, resources, writing – review and editing. **Jacqueline J. Loonen:** data curation, validation, resources, writing – review and editing. **Eline van Dulmen‐den Broeder:** data curation, validation, resources, writing – review and editing. **Helena J. H. van der Pal:** data curation, validation, resources, writing – review and editing. **Saskia M. F. Pluijm:** data curation, validation, resources, writing – review and editing. **Leontien C. M. Kremer:** data curation, validation, resources, writing – review and editing. **Margriet van der Heiden‐van der Loo:** data curation, validation, resources, writing – review and editing. **Marloes Louwerens:** data curation, validation, resources, writing – review and editing. **Hanneke M. van Santen:** data curation, validation, resources, writing – review and editing. **Daniel S. Olsson:** data curation, validation, resources, writing – review and editing. **Imo Hoefer:** data curation, validation, resources, writing – review and editing. **Sjoerd A. A. van den Berg:** data curation, validation, resources, writing – review and editing. **Harm van Tinteren:** conceptualization, methodology, data curation, investigation, validation, formal analysis, supervision, visualization, project administration, writing – original draft, writing – review and editing, resources. **Sebastian J. C. M. M. Neggers:** conceptualization, methodology, data curation, investigation, validation, formal analysis, supervision, visualization, project administration, writing – original draft, writing – review and editing. **Marry M. van den Heuvel‐Eibrink:** conceptualization, methodology, data curation, investigation, validation, formal analysis, supervision, visualization, project administration, resources, writing – original draft, writing – review and editing.

## Conflicts of Interest

D.S.O. is an employee at AstraZeneca as of 30 August 2021.

## Supporting information


**Table S1:** Description of determinants used in analyses.
**Table S2:** Low BMD outcomes (*Z*‐score ≤ −1) using univariable logistic regression analysis.
**Table S3:** Fracture outcomes using univariable logistic regression analysis.

## Data Availability

The data underlying this article were provided by the Princess Máxima Center—DCCSS‐LATER consortium under license. Data will be shared on reasonable request to the corresponding author with permission of the PMC‐DCCSS‐LATER consortium. The data are not publicly available due to privacy or ethical restrictions. Concerning the data of the Lifelines cohort, data may be obtained from a third party and are not publicly available. Researchers can apply to use the Lifelines data used in this study. More information about how to request Lifelines data and the conditions of use can be found on their website (https://www.lifelines.nl/researcher/how‐to‐apply).
