# Supplementary material for: Prevalence and Determinants of Impaired Bone Mineral Density and Fractures in the First National Dutch Wilms Tumor Survivor Cohort, a National DCCSS‐LATER Study
Source: Cancer Med. 2025 Sep 16;14(18):e71229. doi: 10.1002/cam4.71229 (PMC12438959; doi:10.1002/cam4.71229)
Supplement: Supplementary file 1 — Table S1: Description of determinants used in analyses. Table S2: Low BMD outcomes (Z‐score ≤ −1) using univariable logistic regression analysis. Table S3: Fracture outcomes using univariable logistic regression analysis. [file CAM4-14-e71229-s001.docx]

Supplementary Table 1. **Description of determinants used in analyses**

| **Covariate** | **Definition** | **Source** |
| --- | --- | --- |
| **Body mass index** | BMI was calculated using height and weight measurements (height/weight^2^). Weight was adjusted for in case of an amputation. | BMI calculator for amputees; amputee coalition |
| **Body mass index categories** | Underweight: BMI <18.5 kg/m^2^  Normal: BMI ≥18.5 and <25 kg/m^2^  Overweight: BMI ≥25-<30 kg/m^2^  Obese: BMI ≥30 kg/m^2^ | WHO |
| **Heavy drinking** | Males: >14 alcoholic consumptions per week (self-report)  Females: >7 alcoholic consumptions per week (self-report) | NIAAA |
| **Dietary calcium intake** | A survey on weekly consumption was used to determine dietary calcium intake.  Inadequate calcium intake= <3500 g/week. | NEVO-online, RIVM  Mayo Clinic, Nutrition and healthy eating |
| **Physical activity** | The SQUASH survey was used to acquire data on physical activity. The time spent doing moderate-heavy physical activity was compared to Dutch young adults (Lifelines cohort).  Low physical activity=<20^th^ percentile | Wendel-Vos et al.^1^  Lifelines cohort^2^ |
| **Hyperthyroidism** | FT4 levels >24.3 and TSH levels <0.56. FT4 and TSH levels were assessed using the Fujirebio Lumipulse G assay. Of note: FT4 was calibrated to the RMP. The threshold for hyperthyroidism for FT4 was derived from de Grande et al.^3^ TSH was calibrated according to the IFCC harmonization recommendation. The threshold for hyperthyroidism for TSH was derived from Thienpont et al.^4^ | De Grande et al. 2017^3^  Thienpont et al. 2017^4^ |
| **Vitamin D deficiency** | 25OHD levels <50 nmol/L. 25OHD levels were assessed using the Fujirebio Lumipulse G assay. | Manufacturer |
| **Severe vitamin D deficiency** | 25OHD levels <30 nmol/L. 25OHD levels were assessed using the Fujirebio Lumipulse G assay. | Manufacturer |
| **Elevated homocysteine** | Homocysteine levels >19 μmol/L. Homocysteine levels were assessed using the Cobas 6000 c501 assay. | Manufacturer |
| **Vitamin B12 deficiency** | Vitamin B12 levels <150 pmol/L or  Vitamin B12 levels ≥ 150 and <220 pmol/L with elevated homocysteine levels. | UpToDate |
| **Folic acid deficiency** | Folic acid levels <6.8 nmol/L. Folic acid levels were assessed using the Cobas 6000 c601 assay. | WHO |
| Abbreviations: 25OHD=25-hydroxyvitamin D; BMI=body mass index; FT4=free thyroxine; NIAAA=National Institute on Alcohol Abuse and Alcoholism; SQUASH= Short QUestionnaire to ASsess Health enhancing physical activity; TSH=thyroid stimulating hormone; WHO=World Health Organization  ^1^Wendel-Vos GCW, Schuit AJ, Saris WHM, Kromhout D. Reproducibility and relative validity of the short questionnaire to assess health-enhancing physical activity. J Clin Epidemiol. 2003;56(12):1163-1169.  ^2^Scholtens S, Smidt N, Swertz MA, et al. Cohort Profile: LifeLines, a three-generation cohort study and biobank. Int J Epidemiol. 2015;44(4):1172-1180.  ^3^De Grande, LA, Van Uytfanghe K, Reynders D, et al. Standardization of free thyroxine measurements allows the adoption of a more uniform reference interval. Clinical chemistry, 63(10), 1642-1652.  ^4^Thienpont LM, Van Uytfanghe K, De Grande LA, et al. Harmonization of serum thyroid-stimulating hormone measurements paves the way for the adoption of a more uniform reference interval. Clinical Chemistry, 63(7), 1248-1260. | | |

Supplementary table 2**. Low BMD outcomes (Z-score≤-1) using univariable logistic regression analysis**

|  | Low BMD at any site  (n=46/173) | | | Low lumbar spine BMD  (n=34/172) | | | Low total body BMD  (n=25/166) | | | Low total hip BMD  (n=9/73) | | |
| --- | --- | --- | --- | --- | --- | --- | --- | --- | --- | --- | --- | --- |
|  | No. | OR (95% CI) | P-value* | No. | OR (95% CI) | P-value* | No. | OR (95% CI) | P-value* | No. | OR (95% CI) | P-value* |
| Single chemotherapy agents | | | | | | | | | | | | |
| Platinum compounds |  | 2.02  (0.77-5.31) | 0.16 |  | 1.41  (0.48-4.20) | 0.53 |  | NA | 0.49 |  | NA | NA |
| Yes | 8/20 (40.0) |  |  | 5/20 (25.0) |  |  | 4/19 (21.1) |  |  | 0/10 (0.00) |  |  |
| No | 38/153(24.8) |  |  | 29/152(19.1) |  |  | 21/147(14.3) |  |  | 9/63(14.3) |  |  |
| Vinca alkaloids |  | NA | 0.72 |  | NA | 0.48 |  | NA | NA |  | NA | NA |
| Yes | 44/167 (26.3) |  |  | 32/166 (19.3) |  |  | 25/160 (15.6) |  |  | 9/69 (13.0) |  |  |
| No | 2/6 (33.3) |  |  | 2/6 (33.3) |  |  | 0/6 (0.00) |  |  | 0/4 (0.00) |  |  |
| Anthracyclines |  | 2.21  (1.12-4.39) | **0.023** |  | 1.97  (0.92-4.21) | 0.080 |  | 1.88  (0.80-4.42) | 0.15 |  | NA | 0.73 |
| Yes | 26/73 (35.6) |  |  | 19/73 (26.0) |  |  | 14/71 (19.7) |  |  | 5/34 (14.7) |  |  |
| No | 20/100 (20.0) |  |  | 15/99 (15.2) |  |  | 11/95 (11.6) |  |  | 4/39 (10.3) |  |  |
| Alkylating agents |  | 3.19  (1.18-8.63) | **0.022** |  | 1.66  (0.55-5.02) | 0.37 |  | 4.60  (1.50-13.3) | **0.005** |  | NA | NA |
| Yes | 9/18 (50.0) |  |  | 5/18 (27.8) |  |  | 7/18 (38.9) |  |  | 0/8 (0.00) |  |  |
| No | 37/155 (23.9) |  |  | 29/154 (18.8) |  |  | 18/148 (12.2) |  |  | 9/65 (13.8) |  |  |
| Alkylating dose (CED, g/m^2^) |  | 1.02  (0.95-1.09) | 0.64 |  | 1.00  (0.93-1.08) | 0.92 |  | 1.03  (0.96-1.10) | 0.48 |  | NA | NA |
| 0 | 37/155 (23.9) |  |  | 29/154 (18.8) |  |  | 18/148 (12.2) |  |  | 9/65 (13.8) |  |  |
| >0-<8,000 | 6/10 (60.0) |  |  | 3/10 (30.0) |  |  | 5/10 (50.0) |  |  | 0/4 (0.00) |  |  |
| ≥8,000 | 3/8 (37.5) |  |  | 2/8 (25.0) |  |  | 2/8 (25.0) |  |  | 0/4 (0.00) |  |  |
| Cyclophosphamide |  | NA | 0.29 |  | NA | 1 |  | NA | 0.11 |  | NA | NA |
| Yes | 2/4 (50.0) |  |  | 1/4 (25.0) |  |  | 2/4 (50.0) |  |  | 0/3 (0.00) |  |  |
| No | 44/169 (26.0) |  |  | 33/168 (19.6) |  |  | 23/162 (14.2) |  |  | 9/70 (12.9) |  |  |
| Ifosphamide |  | 2.67  (0.91-7.84) | 0.07 |  | NA | 0.50 |  | 3.28  (1.01-10.57) | **0.047** |  | NA | NA |
| Yes | 7/15 (46.7) |  |  | 4/15 (26.7) |  |  | 5/15 (33.3) |  |  | 0/6 (0.00) |  |  |
| No | 39/158 (24.7) |  |  | 30/157 (19.1) |  |  | 20/151 (13.2) |  |  | 9/67 (13.4) |  |  |
| Doxorubicin |  | 1.90  (0.82-4.41) | 0.13 |  | 2.12  (0.87-5.21) | 0.10 |  | 2.85  (1.09-7.47) | **0.034** |  | NA | 0.40 |
| Yes | 11/29 (37.9) |  |  | 9/29 (31.0) |  |  | 8/28 (28.6) |  |  | 3/16 (18.8) |  |  |
| No | 35/144 (24.3) |  |  | 25/143 (17.5) |  |  | 17/138 (12.3) |  |  | 6/57 (10.5) |  |  |
| Epirubicin |  | 1.58  (0.77-3.28) | 0.22 |  | 1.31  (0.58-2.94) | 0.52 |  | 0.77  (0.29-2.07) | 0.60 |  | NA | 1 |
| Yes | 16/48 (33.3) |  |  | 11/48 (22.9) |  |  | 6/48 (12.5) |  |  | 2/20 (10.0) |  |  |
| No | 30/125 (24.0) |  |  | 23/124 (18.5) |  |  | 19/119 (16.0) |  |  | 7/53 (13.2) |  |  |
| Abbreviations: BMD=bone mineral density; BMI=body mass index; CED=cyclophosphamide equivalent dose; CI=confidence interval; NA=not applicable (due to patient numbers <5); No.=number; OR=odds ratio; RT=radiotherapy; Ref=reference  *Logistic regression p-value for variables with more than five observations in each cell. For variables with less than five observations in each cell, a Fisher exact p-value was calculated. | | | | | | | | | | | | |

Supplementary table 3**. Fracture outcomes using univariable logistic regression analysis**

|  | Any fractures  (n=70/221) | | | Long bone fractures  (n=48/221) | | | Fragility fractures  (n=36/221) | | |
| --- | --- | --- | --- | --- | --- | --- | --- | --- | --- |
|  | No. | OR (95% CI) | P-value* | No. | OR (95% CI) | P-value* | No. | OR (95% CI) | P-value* |
| Single chemotherapy agents | |  |  |  |  |  |  |  |  |
| Platinum compounds |  | 1.44 (0.59-3.52) | 0.42 |  | 1.00 (0.35-2.85) | 1.00 |  | NA | 0.38 |
| Yes | 9/23 (39.1) |  |  | 5/23 (21.7) |  |  | 2/23 (8.70) |  |  |
| No | 61/198 (30.8) |  |  | 43/198 (21.7) |  |  | 34/196 (17.3) |  |  |
| Vinca alkaloids |  | NA | 0.38 |  | NA | 0.61 |  | NA | 0.25 |
| Yes | 67/215 (31.2) |  |  | 46/215 (21.4) |  |  | 34/215 (15.8) |  |  |
| No | 3/6 (50.0) |  |  | 2/6 (33.3) |  |  | 2/6 (33.3) |  |  |
| Anthracyclines |  | 0.86 (0.48-1.52) | 0.60 |  | 0.59 (0.30-1.14) | 0.12 |  | 0.63 (0.30-1.32) | 0.22 |
| Yes | 30/100 (30.0) |  |  | 17/100 (17.0) |  |  | 13/100 (13.0) |  |  |
| No | 40/120 (33.3) |  |  | 31/120 (25.8) |  |  | 23/120 (19.2) |  |  |
| Alkylating agents |  | 1.93 (0.79-4.71) | 0.15 |  | 1.40 (0.52-3.80) | 0.51 |  | NA | 0.76 |
| Yes | 10/22 (45.5) |  |  | 6/22 (27.3) |  |  | 4/22 (18.2) |  |  |
| No | 60/199 (30.2) |  |  | 42/199 (21.1) |  |  | 32/199 (16.1) |  |  |
| Alkylating dose (CED, g/m^2^) | | 0.99 (0.94-1.05) | 0.74 |  | 0.99 (0.93-1.06) | 0.84 |  | 0.98 (0.91-1.07) | 0.73 |
| 0 | 60/199 (30.2) |  |  | 42/199 (21.1) |  |  | 32/199 (16.1) |  |  |
| >0-<8,000 | 7/11 (63.6) |  |  | 3/11 (27.3) |  |  | 3/11 (27.3) |  |  |
| ≥8,000 | 3/11 (27.3) |  |  | 3/11 (27.3) |  |  | 1/11 (9.09) |  |  |
| Cyclophosphamide |  | NA | 1 |  | NA | 1 |  | NA | 0.59 |
| Yes | 1/5 (20.0) |  |  | 1/5 (20.0) |  |  | 1/5 (20.0) |  |  |
| No | 69/216 (31.9) |  |  | 47/216 (21.8) |  |  | 35/216 (16.2) |  |  |
| Ifosphamide |  | 1.93 (0.79-4.71) | 0.15 |  | 1.40 (0.52-3.80) | 0.51 |  | NA | 0.50 |
| Yes | 10/18 (55.6) |  |  | 6/18 (33.3) |  |  | 4/18 (22.2) |  |  |
| No | 60/203 (29.6) |  |  | 42/203 (20.7) |  |  | 32/203 (15.8) |  |  |
| Doxorubicin |  | 0.86 (0.40-1.84) | 0.69 |  | 0.63 (0.25-1.61) | 0.33 |  | 0.74 (0.27-2.05) | 0.57 |
| Yes | 11/38 (28.9) |  |  | 6/38 (15.8) |  |  | 5/38 (13.2) |  |  |
| No | 59/183 (32.3) |  |  | 42/183 (23.0) |  |  | 31/183 (16.9) |  |  |
| Epirubicin |  | 0.95 (0.51-1.76) | 0.87 |  | 0.80 (0.39-1.62) | 0.53 |  | 0.71 (0.31-1.61) | 0.41 |
| Yes | 21/68 (30.9) |  |  | 13/68 (19,1) |  |  | 9/68 (13.2) |  |  |
| No | 49/153 (32.0) |  |  | 35/153 (22.9) |  |  | 27/153 (17.6) |  |  |
| Abbreviations: BMD=bone mineral density; BMI=body mass index; CED=cyclophosphamide equivalent dose; CI=confidence interval; NA=not applicable (due to patient numbers <5); No.=number; OR=odds ratio; RT=radiotherapy; Ref=reference  *Logistic regression p-value for variables with more than five observations in each cell. For variables with less than five observations in each cell, a Fisher exact p-value was calculated. | | | | | | | |  | |
